# Introduction to the Special Issue: Ungulates and invasive species: quantifying impacts and understanding interactions

**DOI:** 10.1093/aobpla/plx063

**Published:** 2017-11-09

**Authors:** Bernd Blossey, David L Gorchov

**Affiliations:** Department of Natural Resources, Fernow Hall, Cornell University, Ithaca, NY, USA; Department of Biology, Miami University, Oxford, OH, USA

**Keywords:** Facilitation, herbivory indirect effects, non-consumptive effects, Odocoileus virginianus

## Abstract

White-tailed deer are emblematic ungulates that, due to anthropogenic modification of landscapes, currently occur at elevated densities. Elevated deer densities often co-occur with non-native plants, but it is not known if plant invasions are a consequence of deer impacts or occur independent of deer impacts on ecosystems, or whether these two stressors are synergistic. A colloquium on ‘Interactions of white-tailed deer and invasive plants in forests of eastern North America’ explored these topics at the 2016 annual meeting of the Botanical Society of America. Nine of those presentations are published in this special issue of *AoB PLANTS*.

## Teaser

High densities of white-tailed deer negatively impact ecosystems in parts of North America, many of which are also impacted by invasive plants. Whether plant invasions are a result of high deer population, and how deer and invasives interact in their impacts, are not well understood. This motivated a colloquium at the 2016 Botanical Society of America meeting. Nine of those presentations were expanded for this special issue of AoB PLANTS, “Interactions between white-tailed deer and invasive plants in North American forests.” This Introduction to this issue highlights the context of the problems and summarizes and synthesizes insights from these papers.

## Introduction

Ungulates make up the vast majority of large herbivores, and of the ~257 recognized species (http://www.ultimateungulate.com/ungulates.html) many are well-known charismatic or economically important species including horses, cattle, deer, pigs, giraffes, rhinoceros, goats and camels. Native ungulates around the world share living spaces and resources with many introduced ungulates and domestic livestock on public and private lands. Ungulate populations have fluctuated over time due to climate and hunting pressure, among other factors, and the causes of such changes and the consequences for shaping past and present-day ecosystems have been the focus of much recent ecological work and controversy (Miller *et al.* 2005; Donlan *et al.* 2006; Gill *et al.* 2009; Ripple and Van Valkenburgh 2010; Gill *et al.* 2012).

As ecologists, botanists, conservationists, policy makers or wildlife managers in the 21st century, we are facing, on the one hand, a conservation crisis due to rapid declines and endangerments of ungulate species primarily due to human exploitation (hunting), such as the Saiga antelope (*Saiga tatarica*) in Asia, the African rhinoceros species or the huemul (*Hippocamelus bisulcus*) in South America. On the other hand, populations of other native ungulate species have greatly increased due to human landscape transformations and predator elimination and trophic downgrading of ecosystems (Estes *et al.* 2011) in Europe, Australia, Japan and North America (Côté *et al.* 2004). Additionally, introduced ungulate species have often developed large populations; examples include deer and Himalayan tahr (*Hemitragus jemlahicus*) in New Zealand (Forsyth *et al.* 2010; Foster *et al.* 2014; Cruz *et al.* 2017) and several species of North American and Asian deer in Europe (Fuller and Gill 2001). The problems created by large native or introduced ungulate populations involve not just their direct effects through herbivory (Forsyth *et al.* 2010; Tanentzap *et al.* 2012; Foster *et al.* 2014; Perea *et al.* 2014), but also indirect effects through facilitation of other introduced and invasive species (Vavra *et al.* 2007; Eschtruth and Battles 2009; Nunez *et al.* 2013; Kardol *et al.* 2014; Shelton *et al.* 2014; Dávalos *et al.* 2015*b, c*; Wood *et al.* 2015; Shen *et al.* 2016; Russell *et al.* 2017).

The success of white-tailed deer in North America is a textbook example of wildlife management causing unintended consequences, and hence insights derived from this special issue may be applicable elsewhere. After Europeans arrived in North America, native deer predators (wolves, bears, mountain lions) were mercilessly shot and poisoned. Elimination of large predators was followed by near extinction of deer by recreational and market hunters. Establishment of state wildlife agencies in the early 1900s and conservation measures allowed deer to make a remarkable comeback on a landscape now devoid of their natural enemies, facilitated by forestry practices, agriculture and gardens (Halls 1984). Early local warning signs of negative ecosystem impacts of rapidly increasing deer herds (Leopold *et al.* 1947) were ignored since the return of deer was welcomed by hunters and furthered by the main philosophy of wildlife management agencies to achieve maximum sustainable yield (Jensen 1996; McCall *et al.* 1997; McCullough 2001), a concept quite similar to rangeland management for livestock production (Walker 1995). But this single species focus, and deer browse impacts in forests, gardens, orchards and fields, has resulted in contradictory objectives between those enjoying abundant wildlife (consumptive or non-consumptive interests) and those interested to protect their own fields or gardens or native species that are unable to flourish in the presence of high deer populations.

While experiments and studies across the range of white-tailed deer in North America have for a long time demonstrated that large deer populations create major direct economic (crops, timber, gardens) and ecological problems with impacts affecting entire food webs and ecosystem processes (Côté *et al.* 2004; Wardle and Bardgett 2004; McGraw and Furedi 2005; Nuttle *et al.* 2011; Schweitzer *et al.* 2014), recognition of their indirect, or non-consumptive effects, is rather recent. For example, introduced plants may be symptoms of degradation facilitated by large deer populations (Vavra *et al.* 2007; Knight *et al.* 2009; Kalisz *et al.* 2014) or part of multiple stressor complexes simultaneously affecting many species (Fisichelli *et al.* 2013; Dávalos *et al.* 2015*a, b*). Thus, research on the impacts of invasive plants (e.g. Vila *et al.* 2011; Jauni and Ramula 2015) may yield misleading findings wherever the driver of undesirable changes in ecosystems is deer abundance and invasive plants are merely ‘passengers’ (MacDougall and Turkington 2005). It is also possible that there are synergistic impacts of deer and invasive plants, or that one stressor mitigates the impact of the other (Waller and Maas 2013). It is important to understand these interactions, particularly given that enormous management efforts are directed to lessen impacts of non-native invasive plant species (Foxcroft *et al.* 2014).

### Key insights from the special issue

In August 2016, the Botanical Society of America convened a colloquium on ‘Interactions of white-tailed deer and invasive plants in forests of eastern North America’ at its annual meeting in Savannah, GA. Eleven speakers presented their research findings from studies carried out across the eastern and Midwestern United States. Nine of these investigators have published their findings in this special issue of *AoB PLANTS*.

Two papers in this special issue investigated whether and how deer promote invasion of non-native plants. Heberling *et al.* (2017) tested whether deer herbivory on native herbs promotes invasion of *Alliaria petiolata*, a non-native species avoided by deer (Knight *et al.* 2009). They found that deer herbivory on native herbs increased light availability compared to fenced plots, resulting in a higher maximum photosynthetic rate for *A. petiolata*. While one native herb (*Maianthemum racemosum*) also had greater photosynthetic rate in the presence of deer, this was countered by deer herbivory, and the other native, *Trillium grandiflorum*, had lower photosynthetic rates, as well as browse impacts, in the presence of deer. This study shows how physiological responses mediate the facilitation of *A. petiolata* invasion by high deer populations. Morrison (2017) staged an invasion of *Microstegium vimineum* to test whether deer facilitate the invasion of this annual grass in suburban forests, as well as explore the effects of both drivers on native plants. While fencing did not impede *M. vimineum* invasion, there was a significant interaction of deer and *M. vimineum* on native woody plants: cover of woody plants was lower where deer had access and *M. vimineum* was introduced than in the other treatment combinations.

Three other papers examined combined and potentially interactive effects of deer and invasive plants on native plants. Owings *et al.* (2017) found that both deer and the invasive shrub *Lonicera maackii* reduced survival of seedlings of native oak and American chestnut (*Castanea dentata*) that were ‘underplanted’ in forests. While they found no significant interactions on seedling survival, *L. maackii* removal increased height of chestnut seedlings where deer were excluded but reduced height where deer were present, suggesting this invasive shrub may reduce deer browse on tree seedlings, as indicated by Peebles-Spencer and Gorchov (2017). Similarly, Bourg *et al.* (2017) used a replicated 2 × 2 factorial experiment across three forest stands to investigate effects of excluding deer and removing invasive plants. Deer exclusion had broad impacts on the plant community, resulting in higher native woody species richness and abundance and lower invasive plant abundance. Invasive plant removal had minimal direct effects, but only the combination of deer exclusion and invasive removal resulted in significantly greater native herbaceous richness. Averill *et al.* (2017) provide an integrated set of analyses on data pooled from 23 deer exclusion experiments to tease out deer effects on native and introduced plant species. Overall, deer reduced richness and abundance of native but not of introduced plants, resulting in greater proportions of introduced plants in communities. While certain invasive plant species were facilitated by deer, other invasives that are consumed by deer, including *Rosa multiflora* and *Lonicera* spp., respond rapidly to reduction in deer browse with an increase in cover. These three studies reveal that recovery or enhancement of populations of native plant species will not occur automatically by deer exclusion and removal of certain introduced species may also be warranted where they co-occur. However, invasive plant species removal without deer reduction is certain to fail in most instances or result in minor improvements.

Another paper explored the perspective of invasive plants impacting deer populations. Martinod and Gorchov (2017) provide evidence that the presence and abundance of introduced *L. maackii* establishes an abundant food source for deer that may elevate deer populations in ways that further reduce native plant populations. This effect is analogous to agricultural or ornamental plant subsidies, except that much of the herbivory occurs in early spring, when the invasive shrub has expanded leaves but native woody plants are still leafless. Thus, the direct competition often implied when researchers study effects of introduced on native plants, may instead be apparent competition facilitated by deer.

A second set of three papers examines the collection of evidence for deer impacts. Nuzzo *et al.* (2017) use two different deer reduction methods (fencing and culling) to assess recovery of native plant species. They assess both changes of the community (diversity) and of individuals and demonstrate that while some native plants may recover quickly, the species most threatened by deer show very slow recovery. Furthermore, they document that a focus on individuals in monitoring deer management efforts appears more likely to document benefits than focusing on community metrics like cover or diversity. These findings echo results of a meta-analysis of deer exclusion experiments focused on woody plants (Habeck and Schultz 2015). Blossey *et al.* (2017) introduced a new indicator species approach with repeat visits to marked planted red oak (*Quercus rubra*) individuals and showed that deer exclusion overrides effects of introduced plant species or other mortality factors (such as rodents or insects) in affecting performance of red oaks. This approach shifts the debate from a focus on ungulate numbers to ungulate impact, and this can help evaluate whether the chosen interventions are producing the desired outcomes. At the present time, this ecological assessment of outcomes is not routinely part of management but should be a required, including for management agencies (Decker *et al.* 2016).

Another methodological advance is reported by Erickson *et al.* (2017), who demonstrate a novel way to quantify deer consumption of native and introduced plants using DNA mini-barcoding. By extracting DNA from deer faecal pellets, amplifying the DNA corresponding to a chloroplast gene and comparing DNA sequences of amplified fragments to sequences of plant species in the study area, they were able to assess presence and frequency of plant species in diets of different individual deer. While individual faecal samples revealed each deer fed on numerous plant species, feeding was very selective, with some plants much more common in diets than their availability in the habitat, and others much less common or altogether lacking. Invasive plant species were included in both the set of species over-represented in deer diets (e.g. *Eleagnus umbellata*) and the set of species absent from faecal samples (e.g. *Berberis thunbergii*), indicating that deer preference for invasive plants differs greatly among plant species. This pattern is consistent with evidence from feeding trials (Averill *et al.* 2016).

Emerging from the findings of the studies in this special issue is further recognition of the overwhelming influence of deer in structuring present-day ecosystems and their trajectories, but with the added complication that different invasive plant species may respond differently to high deer populations. Most of the experiments reported in the studies in this special issue were designed and conducted in areas where invasive plant species and deer co-occur to assess their interactions. In these areas, deer exclusion alone will result in population declines of species that are avoided by deer and benefit from exotic earthworms, such as *A. petiolata* and *M. vimineum* which in turn are facilitated by deer (Dávalos *et al.* 2015*c*). However, culling deer or fencing areas where introduced species that are also consumed by deer, such as *Lonicera* spp. and *R. multiflora*, are abundant, may backfire and result in increased cover of these introduced species. Whether such increases are sustained, resulting in suppression of native plants by competition for light and nutrients, or temporary, due to increasing abundance of natives that in turn provide biotic resistance to plant invasion, requires long-term monitoring. While several deer exclosure experiments have been maintained for >10 years (Habeck and Schultz 2015; Averill *et al.* 2017), abundance of invasives has typically only been measured at one or two points in time (Shen *et al.* 2016; Averill *et al.* 2017). More long-term experiments, with periodic assessments, and low (not zero) deer densities are urgently needed. Over much of eastern and Midwestern North America deer reduction may need to be followed by control of invasive plants, at least those eaten by deer, to avoid recruitment problems for native species.

## Conclusions

The contributions in this special issue offer further confirmation for what already is a strong scientific consensus on deer impacts, along with increasing evidence for facilitation of invasive plants by deer, via herbivory or non-consumptive mechanisms. The impacts of deer extend beyond the consequences of direct consumption, leading us to believe that consequences of high deer abundance may have been underestimated and are not yet fully recognized. Despite early warnings (Leopold *et al.* 1947) and decades of accumulating evidence of negative impacts of high deer populations, wildlife management agencies have been unsuccessful in structuring ungulate management in accordance with societal preferences (Jacobson *et al.* 2010; Smith 2011; Bengsen and Sparkes 2016). This failure points to problems in stewardship of natural resources to the benefit of all, not just special interest groups (Hare and Blossey 2014). It appears important to develop a new discourse that incorporates scientific evidence, stakeholder preferences and evidence-based management to develop approaches that safeguard all native species and prevent further erosion of plant biodiversity.

## Sources of Funding

None declared.

## Contributions by the Authors

The general framework of this manuscript was a collaboration by B.B. and D.L.G. The first draft was written by B.B. and revisions were made by both authors.

## Conflicts of Interest

None declared.
